# A new isolation device for shortening gene flow distance in small-scale transgenic maize breeding

**DOI:** 10.1038/s41598-020-72805-x

**Published:** 2020-09-25

**Authors:** Lili Zhang, Shanshan Huo, Yang Cao, Xiang Xie, Yanhua Tan, Yuliang Zhang, Hui Zhao, Pingping He, Jingyuan Guo, Qiyu Xia, Xia Zhou, Huan Long, Anping Guo

**Affiliations:** Institute of Tropical Bioscience and Biotechnology, Key Laboratory of Biology and Genetic Resources of Tropical Crops, Hainan Key Laboratory for Biosafety Monitoring and Molecular Breeding in Off-Season Reproduction Regions, CATAS, Haikou, 571101 Hainan China

**Keywords:** Biotechnology, Ecology, Molecular biology

## Abstract

The transmission of pollen is the main cause of maize gene flow. Under the compulsory labeling system for genetically modified (GM) products in China, isolation measures are crucial. At present, there is no effective isolation device for preventing and controlling the short-range flow of GM maize pollen. The purposes of the present experiments were to overcome the deficiencies of existing technology and to demonstrate a new isolation device for decreasing the gene flow distance of GM maize. The isolation device we invented was shown to be more robust than traditional isolation methods, and it can be disassembled and repeatedly reused. The most important point was that the frequency of gene flow could be greatly reduced using this device. When the distance from the isolation device was more than 1 m, the gene flow rate could be decreased to less than 1%, and when the distance from the isolation device was more than 10 m, the gene flow rate could be reduced to less than 0.1%. When the isolation device was adopted to isolate GM maize in conjunction with bagging the tassels of GM maize at the pollination stage, the gene flow could be controlled to less than 0.1% when the distance from the isolation device was more than 1 m. This device was, however, only applicable for small plots and can shorten the isolation distance of GM maize planting and improve the purity of seeds, all while meeting the needs of close isolation breeding. The use of this device represents a feasible method for risk prevention and control of GM crops.

## Introduction

Maize accounts for 31% of the world's grain production and covers nearly one-fifth of the world's grain-producing land^1^. In addition to its importance as a food crop, fuel ethanol uses maize as the main raw material, and there is genetically modified maize specially used for ethanol production^2^. The United States is the world’s largest producer of fuel ethanol, with an output of about 48 million tons in 2018^3^. In addition, maize straw can also be used in biofuel production^4^. The growth rate of food production is gradually lagging behind that of the global population, which will prompt the need to promote strategies aimed at increasing food production, such as biotechnology research and genetically modified (GM) crop development^5^.

Gene flow refers to the transfer of genetic material (one or more genes) from one biological population to another through a vector^6^. According to the medium, the pathways of gene flow are usually divided into three forms: (1) pollen-mediated gene flow^7–9^, where pollen transmission can be divided into wind pollination and insect pollination^10,11^, (2) seed-mediated gene flow^12^ and (3) propagator-mediated gene flow^13^. The flow distance of wind transmission is generally not more than 150 m. When the breeze is light, the dispersal range of pollen is about 1 m, and when the wind is strong, pollen can disperse 500–1000 m. A field study conducted in the United States estimated that less than 1% of pollen grains would travel more than 60 m. Considering that maize pollen is the largest of food crops, it is not surprising that it is the heaviest wind-borne species in the *Gramineae*. The diameter of maize pollen grains is 103–105 μm^14^, and its sedimentation rate is 0.2–0.3 m/s^15^. Literature studies have shown that an isolation distance of 20 m is enough to make the outcrossing level less than 1%. If outcrossing is required to be less than 0.1%, the recommended distance is more than 100 m^16^. Pollen transmission is the main cause of gene flow, and pollen viability and survival are important factors affecting gene flow. Due to the predictability of the distance, direction and range of pollen flow in wind-borne pollination, the gene flow via wind-borne pollination is relatively predictable, and the efficiency of wind-borne pollination is negatively correlated with the distance between pollen source and recipient plants. In addition, the rates of gene flow in different ecological environments are also very different. It is difficult to estimate the range of gene flow rates from local experimental results^17^.

Gene flow is an objective fact in nature. The factors affecting the gene flow rates of GM maize are very complex. Wind direction, wind speed, distance from the pollen source and pollinating insects are all important factors that cannot be ignored. The source strength of pollen and meteorological conditions at the flowering stage are also major factors determining pollen flow distance in maize. Song^18^ and others suggest that wind speed is one of the main meteorological factors determining pollen diffusion. The maximum pollen diffusion distance is proportional to the wind speed, and the turbulent motion above a canopy has a vital impact on pollen diffusion. Therefore, a certain threshold of pollen flow distance is the result of the combined effect of the two factors, and this may be the reason why different researchers have obtained different safe flow distances for GM maize.

There are two main types of physical restrictions to gene flow. The first type is spatial isolation. There have been studies showing that spatial isolation was an effective measure to control pollen flow under large-scale planting conditions^19^. In China, the obligatory isolation distance between GM and non-GM maize fields is 300 m (China Biosafety Committee). However, it is unlikely that an isolation distance of 300 m is strictly adhered to in real fields, particularly in regions with small-sized fields, as this will limit the choice of freedom of farmers to grow crops^20^. Natural barriers such as mountains, forests and buildings can be used to block the flow of foreign pollen. The other type is the isolation of high-stem crops such as sorghum, sunflower, or hemp by setting up the mesh to block the introduction of exogenous pollen and thus greatly reduce pollen flow^21^. However, gap crops (e.g., barley and sunflower) cannot reduce the frequency of pollen-mediated gene flow between yellow kernel GM maize and non-GM white kernel maize^22,23^. Another mitigation strategy involves time isolation. This uses the adjustment of the planting time of GM and non-GM crops so as to stagger their flowering periods to limit gene flow. In addition, gene flow can be limited by biological restrictions. Unfortunately, most technologies are still in the research stage for limiting gene flow and have not been widely applied in production^24^.

China is currently the only country that uses qualitative labeling. As long as genetically modified components are detected in products, labeling is performed, that is, the labeling threshold is zero. Other countries implement quantitative labeling, that is, threshold management. In most countries, the threshold for a GMO designation is between 3% and 5%, and the threshold for some countries is 1%^25,26^. At present, China adopts the qualitative mandatory labeling system established in 2002 to label 17 types of agricultural genetically modified organisms in five categories; otherwise, they cannot be imported and sold^27,28^. Under the existing zero-tolerance compulsory labeling system for GM foods, the isolation of GM foods from non-GM foods is particularly necessary and important^29^. Under this system, isolation measures are very necessary and even essential; otherwise, when GM crops are planted in large areas, non-GM crops will be more or less affected, and their products will be GM labeled. Growers must choose to either take isolation measures or carry out GM marking. However, conventional reported spatial isolations are far from meeting the requirements for pollen flow limitation in off-season breeding. It is difficult to meet the routine isolation conditions in Hainan during the breeding season, when research and breeding institutions are particularly active. Most maize fields are usually close to each other. Often adjacent fields are less than five meters apart, with few physical obstacles such as trees or fences. At present, there is no effective isolation device in use to prevent and control the short-range flow of GM maize pollen. In order to solve this problem, this study developed a new isolation device for GM maize to control pollen flow. This isolation device combines the actual situation of maize planting systems and production in China and considers the current research hotspots of GM crops to evaluate the ecological safety of GM insect-resistant maize. Finally, our study introduces feasible methods for risk control of GM crops.

## Results

### Gene flow rate statistics of maize at different directions and distances in control and isolation areas

In two planting seasons, it could be seen from the weather forecast for Wenchang City that the main wind direction was northeast in the corn flowering period, and while there were occasional north and east winds, the average wind speed in the flowering period was class 1 (0.3–1.5 m/s) and class 2 (1.6–3.3 m/s).

In the first planting season, the maximum frequency of gene flow in control area A was 12.68% (Table 1). After isolation, the maximum frequency of gene flow in isolation area B decreased to 0.21%, and the frequency of gene flow in the B8 direction was 0 (Table 1). In the second planting season, the maximum frequency of gene flow in control area D was 12.75% (Table 2). After isolation, the maximum frequency of gene flow in isolation area A decreased to 0.025%. In this area, the frequency of gene flow in the A1, A4, A6, A7 and A8 directions was 0 (Table 2). After isolation, the maximum frequency of gene flow in isolation area B was 0 (Table 2). The frequency of gene flow in the B1, B2, B3, B5 and B6 directions was 0 (Table 2), and the maximum frequency of gene flow in isolation zone C was 0.049%. The frequency of gene flow in the C1, C2, C3 and C7 directions was 0 (Table 2).

**Table 1:** Mean (± sd) outcrossing rate at each sample point of the control area and the isolation area in 2016–2017.

Distance	1 m	3 m	5 m	10 m	15 m	20 m	30 m	40 m	50 m	60 m
**Control area A**										
A1	0.18 ± 0.41	0.14 ± 0.44	0.00 ± 0.00	0.00 ± 0.00	0.00 ± 0.00	0.02 ± 0.06	0.00 ± 0.00	0.00 ± 0.00	0.00 ± 0.00	0.00 ± 0.00
A2	5.17 ± 6.84	1.92 ± 2.59	0.04 ± 0.09	0.53 ± 0.55	0.02 ± 0.07	0.31 ± 0.46	0.00 ± 0.00	0.02 ± 0.06		
A3	1.11 ± 1.76	0.25 ± 0.52	2.01 ± 2.46	0.00 ± 0.00	0.00 ± 0.00	0.02 ± 0.07	0.02 ± 0.06	0.00 ± 0.00	0.00 ± 0.00	0.00 ± 0.00
A4	9.65 ± 21.71	2.91 ± 5.05	0.81 ± 1.80	0.04 ± 0.08	0.16 ± 0.19	0.04 ± 0.11	0.00 ± 0.00	0.00 ± 0.00		
A5	0.24 ± 0.26	1.90 ± 4.53	0.61 ± 1.85	0.58 ± 1.58	0.11 ± 0.15	0.08 ± 0.19	0.10 ± 0.21	0.00 ± 0.00	0.00 ± 0.00	0.00 ± 0.00
A6	12.68 ± 21.18	9.89 ± 13.15	1.65 ± 2.74	2.78 ± 4.82	0.05 ± 0.15	0.07 ± 0.16	0.08 ± 0.10	0.00 ± 0.00		
A7	2.34 ± 3.10	0.06 ± 0.18	0.00 ± 0.00	0.10 ± 0.33	0.00 ± 0.00	0.00 ± 0.00	0.00 ± 0.00	0.00 ± 0.00	0.00 ± 0.00	0.00 ± 0.00
A8	0.10 ± 0.17	0.32 ± 0.54	0.00 ± 0.00	0.00 ± 0.00	0.00 ± 0.00	0.00 ± 0.00	0.00 ± 0.00	0.00 ± 0.00		
**Isolated area B**										
B1	0.00 ± 0.00	0.00 ± 0.00	0.21 ± 0.23	0.02 ± 0.06	0.00 ± 0.00	0.00 ± 0.00	0.00 ± 0.00	0.00 ± 0.00	0.00 ± 0.00	0.00 ± 0.00
B2	0.11 ± 0.15	0.19 ± 0.26	0.05 ± 0.10	0.00 ± 0.00	0.00 ± 0.00	0.00 ± 0.00	0.06 ± 0.09	0.00 ± 0.00		
B3	0.02 ± 0.06	0.04 ± 0.08	0.00 ± 0.00	0.04 ± 0.08	0.02 ± 0.06	0.00 ± 0.00	0.00 ± 0.00	0.02 ± 0.05	0.00 ± 0.00	0.02 ± 0.07
B4	0.00 ± 0.00	0.04 ± 0.09	0.00 ± 0.00	0.06 ± 0.17	0.02 ± 0.05	0.00 ± 0.00	0.02 ± 0.05	0.00 ± 0.00		
B5	0.00 ± 0.00	0.07 ± 0.22	0.02 ± 0.05	0.02 ± 0.06	0.02 ± 0.06	0.03 ± 0.08	0.00 ± 0.00	0.00 ± 0.00	0.00 ± 0.00	0.00 ± 0.00
B6	0.19 ± 0.36	0.06 ± 0.13	0.06 ± 0.13	0.04 ± 0.12	0.00 ± 0.00	0.00 ± 0.00	0.02 ± 0.05	0.01 ± 0.03		
B7	0.00 ± 0.00	0.00 ± 0.00	0.02 ± 0.08	0.00 ± 0.00	0.01 ± 0.03	0.00 ± 0.00	0.00 ± 0.00	0.00 ± 0.00	0.00 ± 0.00	0.00 ± 0.00
B8	0.00 ± 0.00	0.00 ± 0.00	0.00 ± 0.00	0.00 ± 0.00	0.00 ± 0.00	0.00 ± 0.00	0.00 ± 0.00	0.00 ± 0.00		

A1–A8 and B1–B8 in the table represent eight directions of NE, N, NW, W, SW, S, SE and E, respectively. The values in the table represent mean (± sd) outcrossing rate at each sample point. The outcrossing rate at each point (1 m, 3 m, 5 m, … 60 m) in the experiment was the mean of the outcrossing rate (P1, P2, P3, … P10) of 10 corn plants at that point.

**Table 2:** Mean (± sd) outcrossing rate at each sample point of the control area and the isolation area in 2017–2018.

Distance	1 m	3 m	5 m	10 m	15 m	20 m	30 m
**Control area D**							
D1	0.03 ± 0.06	0.00 ± 0.00	0.00 ± 0.00	0.00 ± 0.00	0.00 ± 0.00	0.02 ± 0.05	0.00 ± 0.00
D2	2.11 ± 1.73	0.18 ± 0.21	0.43 ± 0.90	0.07 ± 0.09	0.00 ± 0.00	0.00 ± 0.00	
D3	4.26 ± 4.03	0.79 ± 0.49	0.62 ± 0.45	0.31 ± 0.32	0.07 ± 0.13	0.06 ± 0.09	0.04 ± 0.08
D4	12.75 ± 10.27	2.38 ± 0.91	1.17 ± 0.66	0.34 ± 0.30	0.24 ± 0.23	0.38 ± 0.36	
D5	3.98 ± 3.06	0.29 ± 0.16	0.11 ± 0.13	0.02 ± 0.05	0.04 ± 0.08	0.00 ± 0.00	0.02 ± 0.05
D6	0.73 ± 0.54	0.13 ± 0.17	0.18 ± 0.37	0.00 ± 0.00	0.00 ± 0.00	0.00 ± 0.00	
D7	0.02 ± 0.06	0.00 ± 0.00	0.00 ± 0.00	0.00 ± 0.00	0.00 ± 0.00	0.00 ± 0.00	0.00 ± 0.00
D8	0.18 ± 0.41	0.07 ± 0.13	0.00 ± 0.00	0.00 ± 0.00	0.00 ± 0.00	0.00 ± 0.00	
**Isolated area A**							
A1	0.00 ± 0.00	0.00 ± 0.00	0.00 ± 0.00	0.00 ± 0.00	0.00 ± 0.00	0.00 ± 0.00	0.00 ± 0.00
A2	0.00 ± 0.00	0.03 ± 0.08	0.00 ± 0.00	0.00 ± 0.00	0.00 ± 0.00	0.00 ± 0.00	
A3	0.02 ± 0.05	0.00 ± 0.00	0.00 ± 0.00	0.00 ± 0.00	0.00 ± 0.00	0.00 ± 0.00	0.00 ± 0.00
A4	0.00 ± 0.00	0.00 ± 0.00	0.00 ± 0.00	0.00 ± 0.00	0.00 ± 0.00	0.00 ± 0.00	
A5	0.00 ± 0.00	0.02 ± 0.07	0.00 ± 0.00	0.00 ± 0.00	0.00 ± 0.00	0.00 ± 0.00	0.00 ± 0.00
A6	0.00 ± 0.00	0.00 ± 0.00	0.00 ± 0.00	0.00 ± 0.00	0.00 ± 0.00	0.00 ± 0.00	
A7	0.00 ± 0.00	0.00 ± 0.00	0.00 ± 0.00	0.00 ± 0.00	0.00 ± 0.00	0.00 ± 0.00	0.00 ± 0.00
A8	0.00 ± 0.00	0.00 ± 0.00	0.00 ± 0.00	0.00 ± 0.00	0.00 ± 0.00	0.00 ± 0.00	
**Isolated area B**							
B1	0.00 ± 0.00	0.00 ± 0.00	0.00 ± 0.00	0.00 ± 0.00	0.00 ± 0.00	0.00 ± 0.00	0.00 ± 0.00
B2	0.00 ± 0.00	0.00 ± 0.00	0.00 ± 0.00	0.00 ± 0.00	0.00 ± 0.00	0.00 ± 0.00	
B3	0.00 ± 0.00	0.00 ± 0.00	0.00 ± 0.00	0.00 ± 0.00	0.00 ± 0.00	0.00 ± 0.00	0.00 ± 0.00
B4	0.00 ± 0.00	0.00 ± 0.00	0.00 ± 0.00	0.00 ± 0.00	0.01 ± 0.05	0.00 ± 0.00	
B5	0.00 ± 0.00	0.00 ± 0.00	0.00 ± 0.00	0.00 ± 0.00	0.00 ± 0.00	0.00 ± 0.00	0.00 ± 0.00
B6	0.00 ± 0.00	0.00 ± 0.00	0.00 ± 0.00	0.00 ± 0.00	0.00 ± 0.00	0.00 ± 0.00	
B7	0.00 ± 0.00	0.00 ± 0.00	0.02 ± 0.05	0.00 ± 0.00	0.00 ± 0.00	0.00 ± 0.00	0.00 ± 0.00
B8	0.00 ± 0.00	0.02 ± 0.05	0.05 ± 0.06	0.00 ± 0.00	0.00 ± 0.00	0.00 ± 0.00	
**Isolated area C**							
C1	0.00 ± 0.00	0.00 ± 0.00	0.00 ± 0.00	0.00 ± 0.00	0.00 ± 0.00	0.00 ± 0.00	0.00 ± 0.00
C2	0.00 ± 0.00	0.00 ± 0.00	0.00 ± 0.00	0.00 ± 0.00	0.00 ± 0.00	0.00 ± 0.00	
C3	0.00 ± 0.00	0.00 ± 0.00	0.00 ± 0.00	0.00 ± 0.00	0.00 ± 0.00	0.00 ± 0.00	0.00 ± 0.00
C4	0.00 ± 0.00	0.00 ± 0.00	0.00 ± 0.00	0.00 ± 0.00	0.00 ± 0.00	0.02 ± 0.07	
C5	0.05 ± 0.11	0.05 ± 0.06	0.04 ± 0.09	0.00 ± 0.00	0.00 ± 0.00	0.00 ± 0.00	0.01 ± 0.03
C6	0.00 ± 0.00	0.00 ± 0.00	0.00 ± 0.00	0.02 ± 0.05	0.00 ± 0.00	0.00 ± 0.00	
C7	0.00 ± 0.00	0.00 ± 0.00	0.00 ± 0.00	0.00 ± 0.00	0.00 ± 0.00	0.00 ± 0.00	0.00 ± 0.00
C8	0.00 ± 0.00	0.00 ± 0.00	0.00 ± 0.00	0.00 ± 0.00	0.02 ± 0.05	0.00 ± 0.00	

A1–A8, B1–B8, C1–C8 and D1–D8 in the table represent eight directions of NE, N, NW, W, SW, S, SE and E, respectively. The values in the table represent mean (± sd) outcrossing rate at each sample point. The outcrossing rate at each point (1 m, 3 m, 5 m, … 30 m) in the experiment was the mean of the outcrossing rate (P1, P2, P3, … P10) of 10 corn plants at that point.

### Flow distance and frequency of gene flow under different safety thresholds in control and isolation areas

The quality standard of maize seeds is based on the grain crop seed quality standard—Cereals (GB4404.1-2008). Maize seeds are divided into five types: conventional seeds, inbred lines, single-cross, double-cross and triple-cross. These five types have different requirements for seed purity. The 1% threshold refers to an outcrossing rate or gene flow rate less than 1%, i.e., the purity of seeds is more than 99.0%. The 0.1% threshold refers to an outcrossing rate or gene flow rate being less than 0.1%, i.e., the purity of seeds is more than 99.9%. Therefore, considering the needs of commercial production of GM maize and the requirement of hybrid maize seed purity, 1% and 0.1% were selected as allowable thresholds for calculating gene flow distances.

The experimental results from two growing seasons show that under the conditions of no isolation devices, the gene flow rate of maize in the same direction decreased with distance from the pollen donor area. The gene flow rate of maize at 1.0 m was the highest. The maximum frequency of gene flow here was 12–13%, and the gene flow rate of maize in the eight directions at greater than 30 m was very low, almost zero. The farthest distance pollen flow was up to 60 m. Pollen flow could still be found at 60 m, but the gene flow rate was 0.02%, almost negligible (Tables 1, 2), which could meet the requirement of 99.9% purity for seeds. Therefore, after the first season of the experiment, the farthest investigated distance of the gene flow rate was adjusted to 30 m.

In the first planting season (Table 3), the results of the control area showed that when the distance from GM maize was more than 15 m, the gene flow rate could be controlled to less than 1%, and when the distance from GM maize was more than 40 m, the gene flow rate could be controlled to less than 0.1% (Table 1). The gene flow rate of transgenic maize lines in the isolation zone was significantly lower than that of the control. When the distance isolation measure was more than 1 m, the gene flow rate could be controlled to less than 1%, i.e., the purity of seeds could be more than 99.0%, and when the distance isolation measure was more than 10 m, the gene flow rate could be controlled to < 0.1%. The purity of seeds was 99.9% (Table 1).

**Table 3:** Minimum isolation distance of maize plots in 2016–2017 for different safety thresholds.

Different areas	Direction^a^							
	NE	N	NW	W	SW	S	SE	E
**Safe threshold value, 1%**^**b**^								
Control area A	1	5	10	5	5	15	3	1
Isolated area B	1	1	1	1	1	1	1	1
**Safe threshold value, 0.1%**^**c**^								
Control area A	5	30	10	20	40	15	15	5
Isolated area B	10	5	1	1	1	3	1	1

^a^Minimum isolation distance of maize pollen in eight different directions. Positions of distances A1–D8 are shown in Fig. 1.

^b^The minimum isolation distance required by control area A and isolation area B when the gene flow rate reaches the safety threshold of 1% in eight different directions. The units of numbers in the table are meters.

^c^The minimum isolation distance required by control area A and isolation area B when the gene flow rate reaches the safety threshold of 0.1% in eight different directions. The units of numbers in the table are meters.

In the second planting season (Table 4), the results of the control area showed that when the distance from GM maize was more than 10 m, the gene flow rate could be controlled to less than 1%, and when the distance from GM maize was more than 30 m, the gene flow rate could be controlled to less than 0.1% (Table 2). Compared with the control, the gene flow rate of transgenic maize lines in the isolation zone was further reduced. When the distance isolation measure was more than one meter, the gene flow rate could be controlled to less than 0.1%, and the purity of seeds could reach 99.9%. The gene flow frequencies in the three isolation regions A, B and C, all met this standard (Table 2).

**Table 4:** Minimum isolation distance of maize plots in 2017–2018 for different safety thresholds.

Different areas	Direction^a^							
	NE	N	NW	W	SW	S	SE	E
**Safe threshold value, 1%**^**b**^								
Control area D	1	3	3	10	3	1	1	1
Isolated area A	1	1	1	1	1	1	1	1
Isolated area B	1	1	1	1	1	1	1	1
Isolated area C	1	1	1	1	1	1	1	1
**Safe threshold value, 0.1%**^**c**^								
Control area D	1	10	15	n.d	10	10	1	3
Isolated area A	1	1	1	1	1	1	1	1
Isolated area B	1	1	1	1	1	1	1	1
Isolated area C	1	1	1	1	1	1	1	1

*n.d.* not determined.

**Figure 1:**
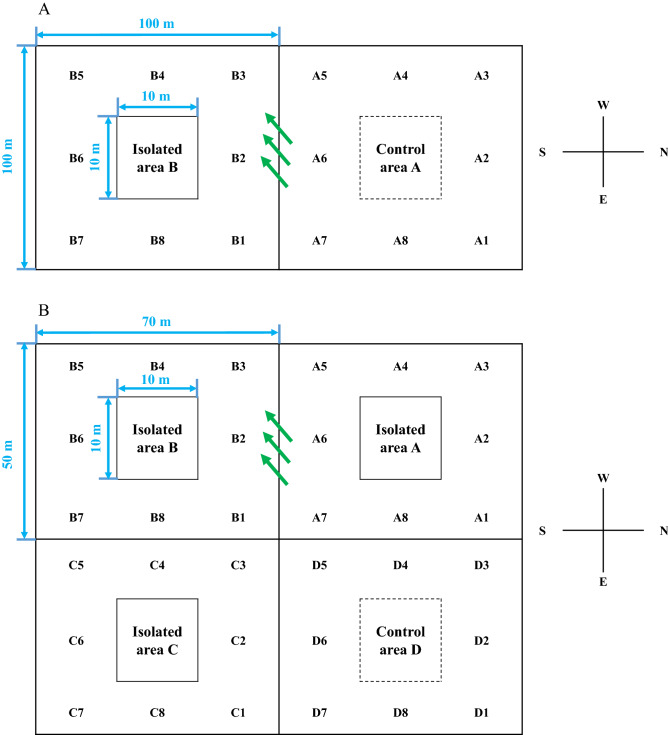
Design of the experimental area. (**A**) In the period of 2016–2017, the design of the experimental area included one control area (A) and one isolation area (B). The dimensions of control area A and isolation area B in the figure are the same. (**B**) In the period from 2017 to 2018, the design of the experimental area included one control area (D) and three isolation areas (A, B and C). The solid line represents the isolation area, and the dashed line represents the control area without isolation devices. A1–A8 and B1–B8 in (**A**) and A1–A8, B1–B8, C1–C8 and D1–D8 in (**B**) represent eight directions of NE, N, NW, W, SW, S, SE and E, respectively. The dimensions of control area D and isolation areas A, B and C in the figure are the same. The blue numbers represent the size of the experimental areas. The green arrows represent the main wind direction during flowering.

^a^Minimum isolation distance of maize pollen in eight different directions. Positions of distances A1–D8 are shown in Fig. 1.

^b^The minimum isolation distance required by control area D and three isolation areas (A, B and C) when the gene flow rate reached a safety threshold of 1% in eight different directions. The units of numbers in the table are meters.

^c^The minimum isolation distance required by control area D and three isolation areas (A, B and C) when the gene flow rate reached a safety threshold of 0.1% in eight different directions. The units of numbers in the table are meters.

The isolation device for natural ecological risk control of GM maize described in this experiment is a colored steel plate. Compared with isolation mesh, the colored steel plate is more solid, convenient to disassemble and can be reused, equivalent to a movable isolation wall. In view of the potential ecological risks of GM crops after planting, this isolation device is of great significance for the safety assessment and control of environment risks of GM crops.

## Discussion

In view of the potential ecological risks of GM maize after planting, it is of great significance to study the safety assessment and control measures for the commercial release of GM maize. Exogenous gene flow mediated by pollen flow or seed dispersal is the main cause of genetic escape. In the prevention and control of maize pollen flow and diffusion, isolation by distance or staggered sowing is generally used to reduce the flow frequency. When the above conditions cannot be met, the general treatment is to set up isolation zones, such as through the planting of high-stem crops, isolation walls or isolation meshes to prevent and control the risk of wind-borne transmission.

The threshold for EU GM identification is 0.9%. In order to meet this threshold, the required isolation distance between Spanish GM maize and traditional maize is 20 m^30^. Studies have shown that an isolation distance of 50 m in Germany can make cross pollination rate less than 0.9%^31^, which is consistent with the isolation distance obtained by Hu et al. (2014)^32^ in the maize planting area of Northeast China. Results from two planting seasons showed that the gene flow rate could be controlled to less than 1% within 20 m of the control area. This was consistent with the study that in the case of a fully synchronous flowering time, a security distance between transgenic and conventional fields of about 20 m should be sufficient to maintain the adventitious presence of genetically modified organisms as a result of pollen flow below the 0.9% threshold in the total yield of the field^33^. Under the existing zero-tolerance compulsory labeling system for GM foods in China, the conventional reported distance isolations are far from meeting the needs of close isolation breeding. The results of two seasons' experiments showed that if the gene flow rate needs to reach the level of less than 1%, it needed to be implemented together with a distance isolation of more than 1 m. If the gene flow rate needs to reach the level of less than 0.1%, it needed to be implemented together with a distance isolation of more than 10 m. When isolation devices and bagging the tassels of GM maize were employed during the pollination period, only a distance isolation of more than 1 m could reduce the gene flow rate to less than 0.1%. This should greatly reduce the isolation distance needed for planting transgenic maize under natural conditions.

Pollen diffusion events mainly occurred in the west, southwest and south, but rarely in the north, northeast and east. This is mainly related to the northeasterly wind prevailing in Hainan Island from September or October to February or March of the following year. According to the meteorological information available for the maize flowering period, the high flow rate area is under the mainstream wind direction in the pollen donor area, while the low flow frequency area is above the mainstream wind direction in the pollen donor area. This is basically consistent with the wind direction and speed that are the main factors affecting pollen diffusion^18,34^.

Our results showed that the frequencies of maize gene flow in eight directions decreased with distance from the pollen donor region. The gene flow rate of maize at 1 m was the highest. Without isolation devices, the maximum frequency of gene flow reached 12–13%. The maximum flow frequency in eight directions above 30 m was 0.01–0.02%, which was far below the threshold requirement of 0.1%. The nearer to the pollen donor area, the greater the difference in flow rate at the same distance in different directions. The farther away from the pollen donor area, the smaller the difference in flow rate at the same distance in different directions. The effect of wind on pollen diffusion decreases with increasing distance from the pollen donor area. This is because the pollen density in the air decreases gradually with the increase in the distance from the pollen donor site^35^. Maize is a wind-pollinated plant. Therefore, the content of airborne pollen decreases with distance from the emitter^36^. Consequently, the out-crossing rate is always highest in the first few meters of a maize recipient field edge facing a maize donor field^37^. This was consistent with the results of many studies, where the frequency of pollen-mediated gene flow from transgenic maize to non-transgenic maize decreased significantly with increasing distance^9^. Cross-pollination rates rapidly declined with distance from a source following a leptokurtic pattern with a long tail, reaching levels below 0.9% farther than 20 m^30^. Maize pollen is relatively heavy for a wind pollinator; although there are many factors that influence pollen dispersal, most pollen will settle down relatively quickly within short distances and will probably not get the chance to interact with most of these factors^38^. When a vertical wind brings pollen out of the isolation device, most of the pollen will also settle down in a short distance, but there will be a parabolic arc during settlement, so the out-crossing rate of 3 m in many directions in the isolation area will be greater than 1 m (Tables 1, 2). The best way to prevent the pollen-mediated gene flow in maize is to set up isolation barriers and add isolation distance.

When sorghum was used as an isolation measure for GM maize, the average rate of gene flow could be reduced from 9.35% to 1.04%, but the sorghum barriers had little effect on the maximum distance of pollen flow, which was closely related to wind direction and speed^39^. The maximum gene flow distance in the control region without isolation and in the region with sorghum isolation was 300 m and 350 m, respectively^39^. The distribution characteristics of wind speed on the leeward side of the protective zone of the maize belt were basically the same as those in the isolation cloth, but the maximum reduction of wind speed on the leeward side of the protective zone of the maize belt was 34–49% on average, which was significantly lower than that of the isolation cloth, which had 64% and 80% on the leeward side of the isolation protective zone. Wind barrier protection plays an important role in controlling pollen diffusion of GM crops. The most important role of wind barrier protection is to reduce wind speed, thereby reducing the risk of wind damage to crops and lessening soil erosion^40,41^. Isolation cloth is a commonly used wind barrier material in the field, but its stability is poor, and basic research is scarce. Technical indicators such as height and distance of a wind barrier have no unified national standards. Yang^42^ used fly netting to control pollen flow. Their results showed that the isolation height should be at least 1.0 m higher than maize when using fly netting to isolate pollen. The higher the isolation netting, the worse the wind resistance. When using screen mesh for isolation, results indicated that screen mesh with a 250 mesh (the pore size of 250 mesh is 58 μm) and height of 2.5 m above the height of transgenic maize was sufficient to prevent gene flow at a critical distance of 20–30 m. This means that the necessary isolation distance to comply with a maximal outcrossing rate of 0.1% can be considerably reduced^43^. When the height of the screen mesh was relatively high, it was found that the screen was easy to be broken or blown down under the strong wind, which was not conducive to the prevention and control of gene flow^43^. After two growing seasons, the new isolation device we invented was shown to be effective to reduce the rate of pollen flow, and the isolation device can be disassembled and reused, making the system equivalent to a movable isolation wall. Most importantly, the system can greatly reduce the rate of pollen flow, shorten the isolation distance during maize planting, improve the purity of seeds and meet the needs for close isolation breeding.

## Conclusion

This isolation device experiment considered the maize planting system in China, the actual production situation, and the current research hotspots of GM crops to evaluate the ecological safety of GM insect-resistant maize. These experiments also studied the possible ecological risks caused by the release of GM insect-resistant maize in the field and explored effective spatial isolation measures of preventing and controlling gene flow and diffusion. After two growing seasons, the new isolation device we invented was shown to be effective to reduce the rate of pollen flow, and this isolation device can be disassembled and reused, making the system equivalent to a movable isolation wall. Most importantly, this system can greatly reduce the rate of pollen flow, shorten the isolation distance during maize planting, improve the purity of seeds and meet the needs for close isolation breeding. This method provides a reference for scientists who need close isolation breeding, provides a reliable experimental basis for the commercialization of GM crops and proposes a feasible method for risk prevention and control of GM crops. To date, this isolation device was tested for small plots of approximately 10 × 10 m. If breeders want to further expand the isolation scale, this device will need further experimental verification.

## Methods

The GM maize material used was the GM insect-resistant maize variety (line) GIF, and the maize was a yellow grain strain provided by the Lai Jinsheng Teacher Laboratory of China Agricultural University. The conventional maize variety Meiyu 11 with white kernels was selected as the pollen receptor of GM maize. The inheritance of the seed (kernel) color can be considered to be a single gene, with one pair of alleles (yellow vs. white). The yellow allele is dominant, and the white allele is recessive. The experimental site was sown at the base of the agricultural GM environmental safety assessment of the Institute of Tropical Biotechnology, Chinese Academy of Tropical Agricultural Sciences, Wujitangxia Village, Maihao Town, Wenchang City, Hainan Province (110° 45′ 44″ E, 19° 32′ 14″ N). Transgenic insect-resistant maize was sown three times, once every other week, so that the pollination period of GM maize overlapped with the silking period of the non-GM maize. Artificial on-demand sowing with three seeds per hole and a 4–5 cm sowing depth was adopted.

Field experiments were carried out during two seasons in 2016–2017 and 2017–2018. In the first planting season of 2016–2017, the farthest investigated distance of flow frequency was 60 m (Fig. 1A, Table 1). According to the results from the first investigation, the frequency of gene flow in the eight directions beyond 30 m was very low, almost zero (Table 1). Thus, in 2017–2018, the farthest investigated distance of flow frequency was adjusted to 30 m. In the second planting season, the total area was approximately 14,000 m^2^ (Fig. 1B, Table 2). As in Hainan off-season reproduction regions the work of breeding research institutes is particularly intensive, it is generally difficult to meet conventional isolation conditions. At the same time, this area also provided a reference for regions around the world that need close isolation. Therefore, we added bagging measures in the treatment areas during the maize tassel pollination period in the second planting season in order to further reduce the flow frequency.

In the first year of the experiment, control and treatment areas were set up. The area of the control region was 10,000 m^2^ (100 m × 100 m). A 100 m^2^ (10 m × 10 m) plot was designated in the center for GM insect-resistant maize, and non-GM maize was planted around this central area. The treatment area with isolation measures was 10,000 m^2^ (100 m × 100 m). A 100 m^2^ (10 m × 10 m) plot was designated in the center for GM insect-resistant maize, and non-GM maize was planted around this area. Colored steel plates were used as an isolation measure. The isolation height was 4 m.

**Figure 2:**
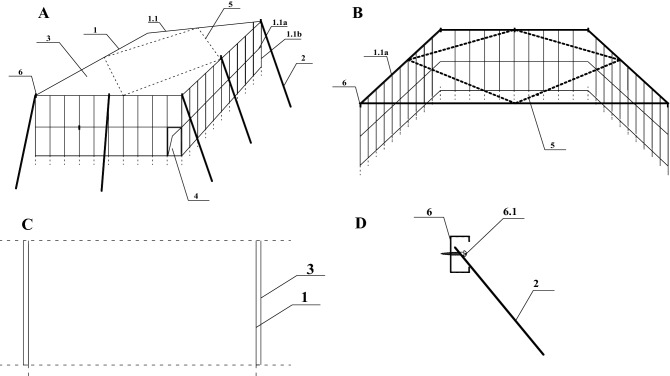
Isolation device for natural ecological risk control of GM maize. (**A**) Schematic of the isolation device; (**B**) partial diagram of the isolation device; (**C**) sectional view of figure (**B**); (**D**) structural detail diagram of the square card; 1: rectangular steel frame, 1.1: steel frame wall, 1.1a: horizontal steel rod, 1.1b: vertical steel rod, 2: inclined support rod, 3: colored steel plate, 4: door for entry and exit, 5: hot-dip galvanized steel frame. 6: structure of the square card, 6.1: screw.

A colored steel plate was the isolation material used in these experiments (Fig. 2). Colored steel plates and steel plates are two different materials. At present, there are many colors of colored steel plates. As for which color was used in our isolation experiments, there was no strict requirement, only a desire to match with the surrounding environment. Colored steel plates have the advantages of having both an organic polymer and a steel plate, and many organic polymers have good colorability, formability, corrosion resistance, decoration and high-strength. This combines with the workability of a steel plate, which can be easily finished by stamping, cutting, bending, deep drawing, and other processing to form virtually any shape. This makes the products made of colored steel plates have excellent practicability, decoration, processing and durability.

When maize was harvested after ripening, the investigated directions of control plots were NE, N, NW, W, SW, S, SE and E, labeled with A1–A8, respectively, and those of the isolation plots were labeled with B1–B8, respectively. The location of GM insect-resistant maize from 1 m, 3 m, 5 m, 10 m, 15 m, 20 m, 30 m, 40 m, 50 m and 60 m was investigated along these eight directions. The farthest investigation distances for NE, NW, SW and SE were 60 m, and other directions were 40 m. Ten maize plants were harvested randomly at each point (the first ear). Plants were marked in the order of P1, P2, P3, … P10, dried and stored for further testing. The total number of kernels harvested per corn ear was recorded.

In the second year of the experiment, one control and three treatments were set up. The control plot and the three treatment areas with isolation measures covered an area of 3500 m^2^ (50 m × 70 m). A 100 m^2^ (10 m × 10 m) plot was designated in the center of the plot to plant GM maize, and non-GM maize was planted around this central area. Colored steel plates were used as an isolation measure. Bagging of tassels of transgenic maize plants was performed during the pollination period. No bagging was conducted in the control area.

When the maize was harvested after ripening, the investigated directions of control plots were NE, N, NW, W, SW, S, SE and E, labeled D1, D2, D3, D4, D5, D6, D7 and D8, respectively. Isolation area A was marked A1, A2, A3, A4, A5, A6, A7 and A8 along the same eight directions. Isolation areas B and C were marked with B1, B2, B3, B4, B5, B6, B7 and B8, and C1, C2, C3, C4, C5, C6, C7 and C8, respectively. The location of GM insect-resistant maize from 1 m, 3 m, 5 m, 10 m, 15 m, 20 m and 30 m was investigated along these eight directions. The farthest investigation distances for NE, NW, SW and SE were 30 m, and the farthest investigation distances for N, W, S and E were 20 m. Ten maize plants were harvested randomly at each point (the first ear). Plants were marked in the order of P1, P2, P3, … P10, dried and stored for further testing. The total number of kernels harvested per corn ear was recorded.

The endosperm was identified by dominant and recessive traits. According to the number of endosperm traits of GM insect-resistant maize harvested at different directions and distances from GM insect-resistant maize, the pollen transmission distance and outcrossing rate of GM insect-resistant maize were then determined. This method can only be applied to dominant endosperm traits such as yellow or non-waxy grains.

The outcrossing rate was calculated according to formula (1):

1 $$ P = \frac{N}{T} \times 100, $$

where P is the outcrossing rate percentage (%), N is the number of corn kernels containing exogenous genes (the number of the yellow seeds) per ear of corn in units of granules, and T is the total grains (the number of the yellow seeds and white seeds) per ear in units of granules. The outcrossing rates of exogenous genes in different directions and distances were determined, and then the pollen flow distance was determined.

As descriptive statistics, the arithmetic mean as well the standard deviation of outcrossing rates were calculated. The outcrossing rate at each point (1 m, 3 m, 5 m, … 60 m) in the experiment was the mean of the outcrossing rate (P1, P2, P3, … P10) of 10 corn plants at that point.

### Details of the isolation device for gene flow risk control of GM maize

The isolation device for gene flow risk control of GM maize, as shown in Fig. 2, comprises a rectangular steel frame (1). The rectangular steel frame 1 was composed of four steel frame walls (1.1), each of which was composed of multiple horizontal steel poles (1.1a) and vertical steel poles (1.1b). Each vertical steel pole was fixed 20–30 cm deep in the soil, and the angle between the inclined support pole (2) and the vertical steel pole was 30°–45°. The vertical steel pole of the four steel frame walls intersected the horizontal steel pole of the top. There were eight inclined supporting poles at the intersection of the vertical steel pole at the four corners of the rectangular steel frame and the horizontal steel pole at the top of the rectangular steel frame, and one inclined supporting pole was fixed through the square card structure (6). The four-sided steel frame wall of the rectangular steel frame was equipped with a colored steel plate (3), and one side of the isolation device was provided with an entry and exit (4). Horizontal steel bars at the top of the rectangular steel frame were provided with a hot-dip galvanized steel frame (5). The hot-dip supporting steel frame was a quadrilateral, and the four corners of the hot-dip supporting steel frame were fixed in the middle of the horizontal steel pole through the hoop. The clamp structure (6) included a side opening and a hollow rectangular frame. The top of the inclined support rod was obliquely inserted into the square clamp structure and fixed on the vertical steel rod through a screw (6.1). The dimensions of the steel rod and the inclined supporting rod were 6000 mm in length, 40 mm in diameter and 2 mm in thickness, and the colored steel plate was 0.425 mm in thickness. The size of the device and the number of inclined supporting rods were determined according to the actual situation in the field.
